# Dying From Dengue Encephalitis in the Absence of DHF: A Case Report

**DOI:** 10.1155/crdi/9852545

**Published:** 2025-10-13

**Authors:** Weerappuli Arachchige Kasuni Suwandika, Vidarsha Rajini Senadeera, Chamara Kiri Vithanage Darshana Ranga, Wijethunga Arachchige Tiron Chathuranga, Janaki Warushahennadi, Arosha Sampath Dissanayake, Chathuranga Lakmal Fonseka

**Affiliations:** ^1^University Medical Unit, Teaching Hospital, Galle, Sri Lanka; ^2^Department of Forensic Medicine, Faculty of Medicine, University of Ruhuna, Galle, Sri Lanka; ^3^Department of Medicine, Faculty of Medicine, University of Ruhuna, Galle, Sri Lanka

**Keywords:** dengue encephalitis, dengue expanded syndrome, dengue fever

## Abstract

**Background:**

Dengue fever is an endemic infectious disease in tropical countries including Sri Lanka with a seasonal transmission. Expanded dengue syndrome consists of unusual manifestations of dengue involving the liver, heart, kidney, and central nervous system. Dengue encephalitis is a rare neurological manifestation of expanded dengue syndrome which can lead to significant morbidity and mortality. Among patients with dengue encephalitis, rapid deterioration without plasma leakage and preceding neurological symptoms and signs is an unusual presentation.

**Case Presentation:**

We report an 18-year-old previously healthy male, who presented with fever for three days with mild frontal headache without other preceding neurological signs who succumbed following rapid deterioration and generalized tonic-clonic seizures complicated with status epilepticus despite optimal medical management. He did not have features of plasma leakage clinically and through focused ultrasound and did not have rising pack cell volume. He was positive for dengue NS1, IgM, and RT-PCR for dengue Serotype 3 with histopathological findings of the brain revealing dengue encephalitis.

**Conclusions:**

Dengue encephalitis can lead to sudden and unexpected mortality associated with status epilepticus with or without signs of dengue hemorrhagic fever. It is a rare manifestation seen in dengue expanded syndrome and can cause sudden death without warning symptoms and signs. The initial presentation may have little warning signs without obvious neurological signs; hence, it is imperative to be vigilant on atypical but potentially lethal presentations of dengue fever.

## 1. Background

Dengue is an arthropod-borne viral disease transmitted by Aedes mosquitos. Dengue fever is caused by the single-stranded RNA Flavivirus genus which consists of four different antigenically related but distinct dengue virus serotypes [1]. It is estimated to cause 390 million dengue infections per year, of which 96 million cases were spread in nearly 129 countries around the world [2]. Apart from the underassessed burden of asymptomatic illness, the patients presents with dengue fever, dengue hemorrhagic fever, and expanded dengue syndrome (EDS) [3]. The World Health Organization (WHO) introduced the term EDS incorporating a wide spectrum of unusual manifestations during dengue viral infection raging from a mild self-limiting illness to a disease leading to fatality [3]. In the recent years, with the wider geographical spread of dengue, there has been an increasing incidence and greater emphasis on EDS.

EDS represents cases of dengue which do not fall into either dengue shock syndrome or dengue hemorrhagic fever [4]. The manifestations of expanded dengue include patients with dengue fever with unexpected organ involvement such as the liver, heart, kidney, and central nervous system [1]. Dengue encephalitis is a neurological complication of dengue viral infection. It is a manifestation of EDS which can cause severe damage to the central nervous system [4]. Dengue is not classically a neurotropic virus, although there is recent evidence of direct neuronal injury [5]. Pathogenesis of neurological manifestations of dengue viral infection remains poorly understood. It may be due to direct invasion of the central nervous system, neurotrophic effects, autoimmune-mediated mechanisms, or secondary to metabolic derangements [6]. Clinical and laboratory manifestations of dengue encephalitis may be characterized by altered level of consciousness, seizures, or personality changes with positive NS1 antigen, reactive IgM antibody, or dengue PCR in CSF or serum [7, 8]. Neurological manifestations such as transverse myelitis, myositis, and Gullain–Barre syndrome have been more commonly reported [9]. Herewith, we report an unusual case of dengue encephalitis who succumbed despite supportive and resuscitation measures.

## 2. Case Presentation

An 18-year-old boy presented with a 3-day history of high-grade fever associated with arthralgia, myalgia, one episode of vomiting, headache along with retro-orbital pain, and postural dizziness. On Day 3, he was admitted to the hospital. Headache was frontal, mild in severity, and did not worsen during the course of his illness. He did not have any episodes of confusion or abdominal pain. On examination, he was afebrile, pulse 80 bpm, blood pressure of 100/70 mmHg, capillary refilling time less than 2 second, and oxygen saturation of 100% on room air. The respiratory examination was normal with no pleural effusion. His abdominal examination revealed mild right hypochondrial tenderness with no hepatosplenomegaly. The rest of the systemic examination was normal. He had a Glasgow coma score of 15/15 with no neck stiffness and no evidence of a focal neurological deficit. On admission, he had a WBC of 2.8 × 10^9^/L, lymphocytes 0.7 × 10^9^/L, hemoglobin 15.3 g/dL, and platelet count of 1,240,00 × 10^9^/L. Capillary blood was extensively used to monitor bedside hematocrit in all inward dengue patients. His initial capillary blood packed cell volume (PCV/Hct) was 40%, and bed side ultrasound scan of the abdomen revealed no free fluids in lungs, hepatorenal pouch or rest of the abdomen.

On Day 4, his white cell counts gradually dropped down to 2.38 × 10^9^/L and platelet count to 56,000 × 10^9^/L with static PCV. His aspartate transaminase level was repeatedly around 92 U/L, and alanine transaminase level was 29 U/L. He was managed as dengue fever in the precritical phase and monitoring was performed and managed according to the national guidelines of Sri Lanka [10]. After platelet count dropped below 100 × 10^9^/L, we performed bedside ultrasound of the chest and abdomen twice daily which did not show any evidence of leaking or gallbladder wall edema. A focused ultrasound scan was performed 2 h before he deteriorated. He did not have any warning signs of severe disease such as abdominal pain, dizziness, or vomiting.

On Day 4, at night he collapsed near his bed and was found to have a reduced level of consciousness. His eyes rolled up and had generalized rigidity with tonic-clonic movements. His airway was patent with respiratory rate of 30 breaths per minute and SpO_2_ of 100%. He was tachycardic with 150 bpm, had a regular thready pulse and a blood pressure of 70/50 mmHg. His GCS level was 6/15 with dilated equally reactive pupils of 4 mm, and his capillary blood sugar level was 151 mg/dL. His airway was secured using an oropharyngeal airway, resuscitated with 0.9% saline fluid boluses, and the blood pressure rose to 90/60 mmHg. He was given IV levetiracetam loading dose and then IV phenytoin loading dose and infusion. Due to lack of clinical response, he was started on IV midazolam infusion. His ECG revealed sinus tachycardia, and VBG showed pH-7.35, pCO2-28, pO2-44, HCO_3_-15.5 and lactate of 6.9. He was intubated and ventilated. Due to persistent hypotension, he was initiated on IV noradrenaline infusion. Despite all efforts, he continued to have generalized tonic-clonic seizures indicative of status epilepticus. Profuse bleeding from oral cavity and cannula sites was noted, and he was managed as for disseminated intravascular coagulation. Later, he had a cardiac arrest, and we continued cardiopulmonary resuscitation. After 10 cycles, we could achieve return of spontaneous circulation (ROSC). Venous blood gas which we performed after ROSC revealed lactic acidosis (pH-7.12, lactate −10.9, HCO_3_ 10.9). His metabolic parameters were promptly corrected. On Day 5, early morning, he had another cardiac arrest after 30 min and cardiopulmonary resuscitation was restarted, but he succumbed after an hour. Due to the rapid deterioration of the patient lumbar puncture, EEG and MRI brain were not performed.

**Figure 1:**
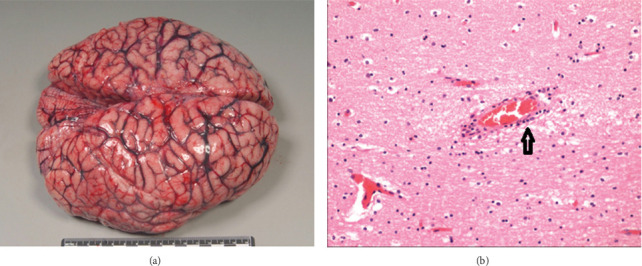
Macroscopic and microscopic appearance of the brain of the patient. (a) Specimen showing gross cerebral edema with congested vessels. (b) Histology shows perivascular cuffing with lymphocytes.

He had positive dengue NS1 antigen and IgM performed on Day 4 of illness (dengue IgG was negative). Blood samples detected dengue Serotype 3. Postmortem examination revealed a significant cerebral edema with congested blood vessels. The weight of the brain was 1400 g (Figure 1(a)). The lungs were congested with numerous petechial hemorrhages on the visceral pleural surfaces. The myocardium was pale, flabby with flame-shaped subendocardial hemorrhages (not shown). Histological findings of brain tissues showed marked cerebral edema with perivascular cuffing with lymphocytes suggesting the presence of encephalitis (Figure 1(b)).

## 3. Discussion

EDS is an under-reported entity that ranges from mild to severe disease manifestations. We report a young man who succumbed due to dengue encephalitis. He did not have any warning signs such as persistent vomiting, abdominal pain, bleeding manifestation, or features of impending shock such as postural dizziness, postural or resting hypotension, reduction of pulse pressure throughout the admission, and did not have confusion. The patient was assessed routinely 2 h before he collapsed, and specific signs of encephalitis or any other warning signs were not evident. His vitals were normal, and the focused bed-side ultrasound scan by a trained doctor did not show any evidence of leaking. This signified that the patient did not have any warning sign of severe dengue disease, DHF, or dengue shock. Currently, no specific treatment exists for dengue or dengue hemorrhagic fever except for meticulous fluid management. In addition to giving maintenance fluid volume, EDS does not have any specific treatment modalities.

Status epilepticus was triggered due to dengue encephalitis, where he was treated with intravenous benzodiazepines, phenytoin, and levetiracetam. In addition, evidence suggests that lacosamide or brivaracetam can be used as adjunctive treatment. In addition, intravenous ketamine, midazolam, and propofol infusion can be used as subsequent therapy for refractory status epilepticus; however, this could not be used due to the lack of availability and due to rapid deterioration.

According to WHO, EDS is defined as unusual manifestations of dengue with various systemic manifestations. A recent study revealed that the gastrohepatic system was affected in majority (92%) followed by CNS involvement (5.5%). Of those with EDS with gastrohepatic involvement, the majority had asymptomatic elevation of liver enzymes followed by those with features of a calculous cholecystitis [11]. Some of them admitted with acute abdomen. A few had acute kidney injury requiring hemodialysis [11].

The central nervous system involvement was the second most common EDS manifestation being present in 5.5%. The pathogenesis of neurological manifestations of dengue infection is yet to be understood. Manifestations occur in brain parenchyma, brain stem, or spinal cord causing encephalitis, meningitis, and myelitis [12–17]. There are reported cases of involvement of the central nervous system, eyes, and peripheral nervous system possibly due to direct viral invasion or postdengue immune-mediated mechanism [18, 19]. Dengue encephalitis is a rare occurrence, which is thought to be secondary to direct viral invasion occurring mainly in the viremic phase, as seen in this patient. A study demonstrated the presence of dengue antigen in brain tissue, neurons, astrocytes, microglia, and endothelial capillary cells by immunohistochemistry using antibody specific for dengue [13]. The usual interval between the systemic features and neurological manifestations of dengue is three to 7 days. Typical features of dengue encephalitis include fever, headache, altered sensorium, seizures, and focal neurological deficit, in the absence of any metabolic abnormalities [20]. Our patient had an ongoing fever and mild sleepiness on Day 4 of illness and later progressed to status epilepticus and shock. Unfortunately, there were no warning signs of severe disease during his illness.

Dengue encephalitis is proposed to be defined as detection of dengue virus RNA, IgM, or NS1 antigen in CSF and CSF pleocytosis without other neuroinvasive pathogens [21]. Our patient had clinical syndrome of dengue encephalitis along with confirmatory serological investigations and PCR with histopathological finding of encephalitis. A previous case report of dengue encephalitis, from Sri Lanka described a case of dengue encephalitis in dengue critical phase, where the presentation was associated with high grade fever, left upper limb and lower limb tonic-clonic convulsions, followed by secondary generalization and persistent weakness of the left side of the body [22]. Another case report from Sri Lanka reported a 14-year-old boy presented with generalized tonic-clonic seizures lasting a few minutes on third day of fever with no other neurological deficits [23]. In both these case reports of dengue encephalitis, the patient was in dengue ‘critical phase' (had vascular leakage) and opposed to our patient who did not have any features of leakage. In another case described by Madi et al., the patient was presented on Day 6 fever with headache with altered consciousness later developed a GTC seizure. The case did not specifically mention whether the patient developed vascular leakage [24]. These reported patients had neurological symptoms and signs on presentation such as headache, seizures, coma, and weaknesses which we did not encounter in this patient during his course of illness before deterioration to status epilepticus [1, 22, 25]. In the cases reported above [22, 23], EEG findings showed slow waves and generalized spike activity and periodicity suggestive of encephalitis, respectively. Weerasinghe et al. reported although serum dengue IgM was positive, the CSF serology was negative initially; however, dengue IgG in CSF was positive in the follow-up visit [22]. Weerarathna et al. reported the CSF positivity for dengue IgM [23]. The imaging studies carried out on the abovementioned cases included MRI brain findings of abnormal high intensity subcortical white matter and cortical gray matter in right frontoparietal and temporal lobes in T2-weighted (T2W) and fluid-attenuated inversion recovery (FLAIR) images with some faint meningeal enhancement appreciated in right frontotemporal area suggestive of meningoencephalitis [22, 23]. In addition, a 23-year-old woman presented with hemodynamic shock with ultrasonic evidence of mild plasma leakage and widespread neurological involvement. MRI imaging revealed extensive midbrain, cerebral, and cerebellar changes consistent with acute dengue encephalitis [25]. The mortality associated with dengue encephalitis is high as several published cases reported mortality and significant disability [26, 27]. Some cases showed features of acute hemorrhagic dengue encephalitis [20, 28], and one had high signal intensity area in bilateral thalamus, pons, medulla, and left cerebellum with intense restriction resembling a double doughnut sign [29] (Table 1).

According to the literature, autopsy studies reported on fatal cases of dengue with neurological involvement showed histopathological patterns of cerebral edema, congestion, hemorrhage, perivascular neutrophilic or lymphocytic infiltration, and even hemorrhagic areas. There was also parenchymal edema and microinfarcts throughout the midbrain, pons, medulla, cerebellum, cerebral cortex, and dura [31]. Petechial hemorrhages in congested lungs could occur due to vigorous resuscitation, and subendocardial hemorrhages were due to shock following cardiac arrest in our patient according to local forensic pathologist's opinion. Currently, the mortality rate in dengue encephalitis is reported to be around 33% from studies conducted in India and Pakistan [26]. Much is understood about pathophysiology, clinical manifestations, and optimal management of DHF, and further studies are required to acquire the same understanding about EDSs with their different manifestations, pathologies, and management strategies.

## 4. Conclusion

Dengue encephalitis can lead to sudden and unexpected mortality in patients with dengue fever with or without features of dengue hemorrhagic fever. The initial presentation may have little warning signs and without obvious neurological signs suggestive of central nervous system involvement. It is imperative to be vigilant on atypical but potentially lethal presentations of dengue fever.

## Figures and Tables

**Table 1:** Clinical presentation, investigations, and outcome of published cases of dengue encephalitis.

Demographic details	Presentation	Investigations	Outcome
37, female [20]	Fever, chills, altered mentation, vomiting, GCS-8/15, and plasma leakage not mentioned specifically	Thrombocytopenia, transaminitis, NCCT-diffuse cerebral edema, CSF analysis had normal protein and glucose levels, no pleocytosis, and an absence of oligoclonal bands, MRI-acute hemorrhagic necrotizing encephalitis (frontal, temporal, cerebellar,and thalamic)	Developed bilateral horizontal gaze palsy, dysarthria, and truncal ataxia. Discharged
27, male [20]	Poor sensorium, three episodes of generalized tonic-clonic seizures-6 h, hypotension, and plasma leakage not mentioned specifically	Thrombocytopenia, transaminitis NCCT-ill-defined hypodensity in thalami, cerebellar and medulla. Repeat NCCT-diffuse cerebral and cerebellar edema with intrathalamic bleeding, CSF-normal protein, and glucose levels, no pleocytosis	Succumbed
18, male [22]	Fever, left hemiparesis, tonic-clonic convulsions, horizontal gaze palsy, GCS 10/15, plasma leakage present	Leukopenia, thrombocytopenia, CSF-lymphocytes 3/mm^3^, sugar-normal, protein-250 mg/L, EEG performed on the following day showed generalized slow waves, severe hepatitis, NCCT & CECT normal, `MRI- right frontal and temporal high intensity subcortical white matter and cortical gray matter	Full recovered on discharge
14, male [23]	Fever, generalized tonic-clonic seizures, plasma leakage present	Leukopenia, thrombocytopenia NCCT-normal, EEG-generalized spike activity and periodicity, CSF-normal, CSF dengue IgM positive	Full recovery
49, M [24]	Fever, headache, altered sensorium, generalized tonic-clonic seizures, mild hepatitis	MRI brain-normal, EEG-slow waves, CSF-80 lymphocytes/μL	Fully recovered
17, female [27]	Fever, rigors, headache and altered sensorium, generalized tonic clonic seizure with unresponsiveness, hypotension/shock, evidence of leaking not mentioned.	Thrombocytopenia, transaminitis, CSF-mildly raised protein and lymphocytic, serum and CSF positive IgM antibodies MRI- bilateral cerebellar cortex, vermis of the cerebellum, pons, midbrain, bilateral medial temporal lobes, and both thalami	Succumbed
23, female [30]	Fever, altered sensorium, GCS 3/15, hypotensive, low oxygen saturation, plasma leakage present	Thrombocytopenia, anemia, transaminitis. MRI brain—acute necrotizing meningoencephalitis	Alive but only slight improvement in her neurological status
13, female [28]	Fever, headache, vomiting. Generalized tonic clonic seizure, mild plural effusions, plasma leakage present	MRI - asymmetric hypodensity in bilateral deep gray matter nuclei and right posterior parietal lobe. CSF - mildly elevated protein with lymphocytosis. Serology and CSF for dengue (IgM and IgG) positive. Repeat MRI was performed which revealed hemorrhages.	Improved fully
18, female [29]	Fever, vomiting, quadriparesis, dysarthria. Bilateral horizontal gaze palsy with impaired oculocephalic reflex, bulbar dysarthria and quadriplegia with bilateral planters up-going, plasma leakage present	CSF analysis revealed normal cell counts and slightly elevated protein levels. The patient had a positive serum NS-1 antigen. MRI - bilateral thalamus, pons, medulla, and left cerebellum, double doughnut sign	Improving, partial recovery on discharge

## Data Availability

The data that support the findings of this study are available from the corresponding author upon reasonable request.
